# AI-Native PHY-Layer in 6G Orchestrated Spectrum-Aware Networks

**DOI:** 10.3390/s25237206

**Published:** 2025-11-26

**Authors:** Partemie-Marian Mutescu, Adrian-Ioan Petrariu, Eugen Coca, Cristian Patachia-Sultanoiu, Razvan Marius Mihai, Alexandru Lavric

**Affiliations:** 1 Faculty of Electrical Engineering and Computer Science, Ștefan Cel Mare University of Suceava, 720229 Suceava, Romania; marian.mutescu@usm.ro (P.-M.M.); apetrariu@usm.ro (A.-I.P.); eugen.coca@usv.ro (E.C.); 2 Orange, 010665 Bucharest, Romania; cristian.patachia@orange.com (C.P.-S.); razvan.mihai@orange.com (R.M.M.)

**Keywords:** 6G networks, artificial intelligence, B5G, spectrum sensing

## Abstract

The evolution from fifth generation (5G) to sixth generation (6G) networks demands a paradigm shift from AI-assisted functionalities to AI-native orchestration, where intelligence is intrinsic to the radio access network (RAN). This work introduces two AI-based enablers for PHY-layer awareness: (i) a waveform classifier that distinguishes orthogonal frequency-division multiplexing (OFDM) and orthogonal time frequency space (OTFS) signals directly from in-phase/quadrature (IQ) samples, and (ii) a numerology detector that estimates subcarrier spacing, fast Fourier transform (FFT) size, slot duration, and cyclic prefix type without relying on higher-layer signaling. Experimental evaluations demonstrate high accuracy, with waveform classification achieving 99.5% accuracy and numerology detection exceeding 99% for most parameters, enabling robust joint inference of waveform and numerology features. The obtained results confirm the feasibility of AI-native spectrum awareness, paving the way toward self-optimizing, context-aware, and adaptive 6G wireless systems.

## 1. Introduction

Mobile communication systems are rapidly changing due to the increase in the number of connected devices, the variety of the offered services, and the requirements imposed by new applications [1]. Compared to the fourth generation (4G) of mobile communications, also known as Long-Term Evolution (LTE), the fifth-generation (5G) networks brought several improvements in terms of spectrum efficiency, data transmission rates, and latency. Following 5G, the sixth generation (6G), which is currently under development, is seen as an advanced communication platform that is based on artificial intelligence (AI), being adaptive and fully cognitive [2,3]. Considering this, compared to previous generations, where network operation was based on predefined procedures and static resource allocation, 6G will require orchestration across the communication, sensing, and computation layers in order to obtain the expected performance level.

In this context, AI is expected to be the main enabler of the future 6G networks, driving both the Core Network and RAN (Radio Access Network), rather than being limited to isolated tasks such as traffic prediction or radio interference mitigation. Additionally, AI is expected to coordinate multiple network functions in 6G, ensuring reliable operation across a wide range of communication scenarios. The main concept supporting the future 6G vision is the AI orchestrator, an entity formed of connected task-specific AI algorithms that aim to manage the network from one end to another across all layers. This AI-based orchestrator dynamically allocates radio spectrum resources, optimizes Core to RAN operations, and adapts configurations in real time [4].

Compared to previous implementations of AI techniques in wireless communications, the AI orchestrator in 6G networks is expected to be the next evolutionary step to autonomous, cognitive, and virtualized infrastructures. In earlier generations of mobile communications, AI techniques were applied to specific tasks like channel estimation, interference reduction, and traffic prediction. In contrast, the 6G AI orchestrator will function as a system-wide intelligence. It will coordinate all layers, from the core network to the radio frontend, including radio spectrum management and resource allocation optimization at the PHY layer. It will also enhance security, resilience, and adaptability under changing and challenging conditions in the upper layers of the network. Considering this, the AI orchestrator becomes the central enabler of the future 6G networks, for ultra-reliable low-latency communications (URLLC), massive machine-type communications (mMTC), and augmented reality services.

A key function of the future 6G networks will be radio spectrum awareness. Traditional spectrum sensing techniques are usually employed to determine if a communication channel is free or occupied. However in 6G networks, spectrum sensing will become a contextual awareness capability that provides advanced communications channel analytics such as radio waveform type, numerology schemes, modulation type, interference patterns, and even environmental context [5]. In the context of 5G, numerology refers to a set of PHY-layer parameters that define the configuration of the Orthogonal Frequency Division Multiplexing (OFDM) waveform. This includes subcarrier spacing (SCS), FFT size, slot duration, and cyclic prefix (CP) type. Compared to LTE, which used a fixed OFDM waveform configuration of 15 kHz SCS and normal CP [6], 5G networks use scalable numerologies with SCS values of 15 kHz, 30 kHz, 60 kHz, 120 kHz, and up to 240 kHz [7]. Each numerology is suitable for different communication scenarios; for example, lower SCS values with longer symbol durations provide better coverage in wide-area deployments, while higher SCS values are used in low-latency, high-capacity applications in FR2 (Frequency Range 2) mmWave bands or high-mobility environments due to the higher Doppler tolerance. The flexibility of the OFDM waveform parameters enables dynamic resource allocation. Multiple numerology schemes can exist in the same frequency bands. These schemes can be adjusted according to service needs and interference conditions [8].

For 6G, this flexibility is further extended, as both gNodeBs (gNBs) and User Equipment (UE) must not only identify the numerology of incoming waveforms, but also adaptively switch between them as the communication scenario is changed. The spectrum awareness features allow waveform flexibility. UEs and gNBs can change modulation, frame structures, and numerology schemes based on channel conditions, latency needs, or the presence of multiple services in the same frequency band. Therefore, the use of an AI orchestrator becomes mandatory for managing all these decisions in the network, ensuring that multiple numerologies can coexist, while monitoring spectral efficiency in cell-free and AI-native network architectures.

Besides flexible and scalable OFDM numerologies, next-generation mobile networks are also evaluating new waveforms for high-mobility situations. The best candidate for this application is Orthogonal Time Frequency Space (OTFS) modulation [9]. OFDM waveforms place symbols onto the time–frequency grid, which can handle small Doppler shifts. However, OFDM waveforms experience performance issues with large Doppler shifts. These situations frequently occur in advanced mobility settings like high-speed vehicular networks, unmanned aerial vehicle (UAV) communications, and low-Earth orbit (LEO) satellite links. All of these are important use cases for 6G. Compared to OFDM, in OTFS waveforms the symbols are mapped in the delay-Doppler domain. This approach maintains channel stationarity and allows equalization in rapidly varying channels. Additionally, recent studies [10] have shown that OTFS can enhance Integrated Sensing and Communication (ISAC) performance by reducing pilot-to-data and intercarrier interference. Considering the advantages and waveform characteristics of OTFS, they become a complement to OFDM in future 6G systems, making their detection by spectrum sensing techniques an important part of waveform agility in AI-driven orchestration.

In addition to OFDM and OTFS, compact waveform designs can be used for fragmented spectrum use, which increases the spectrum efficiency and coexistence of multiple services in the same frequency band [11]. Therefore, the ability to correctly identify and adjust to the waveform and its parameters becomes an important part of future 6G networks.

The use of AI at the RAN level improves its capabilities by allowing data-driven processing of the PHY layer. Deep learning (DL) models can extract signal features directly from raw In Phase/Quadrature (IQ) samples without depending on traditional signal processing techniques. The use of AI techniques in wireless communications has been shown to achieve a high level of performance in multiple spectrum sensing applications. These include channel state identification, modulation recognition, and anomaly detection. The accuracies of these applications exceed 90%, even in low SNR conditions. In our earlier works, AI models have successfully handled tasks such as LoRa modulation detection, advanced channel analytics for IoT communications [12] and LTE and 5G communication detection [13], confirming the further usage of these methods beyond 5G. By transferring these approaches to future 6G networks with AI orchestration, scalable frameworks where AI models can generalize across multiple waveforms and frequency bands can be developed, making AI a key enabler for cognitive orchestration in future cell-free network deployments. By processing raw IQ samples directly, AI-based spectrum sensing techniques avoid the need for higher-layer signaling in the decision-making process inside the network, even in low SNR and high interference environments [14]. Their role within the AI orchestrator goes beyond PHY-layer sensing. The data from external sensing can improve scheduling, UE handover, interference reduction, and anomaly detection. Together, these abilities allow cell-free architectures to coordinate distributed access points (APs) and keep service quality consistent.

This work addresses two aspects of the future 6G network AI orchestrator. First, it introduces an AI-based waveform type detector capable of distinguishing between OFDM and OTFS signals directly from raw IQ samples. By differentiating the two waveform types, receivers can set up the right demodulator chain. This reduces synchronization errors and allows for transitions between waveforms based on the communication scenario. Second, an AI-based OFDM numerology scheme detector that goes beyond waveform type identification by extracting the OFDM waveform configuration parameters such as SCS, FFT size, slot duration, and CP type. Instead of viewing numerology as fixed settings, the detector changes them into dynamic features of the PHY layer that can be inferred directly from the received radio signal without applying any signal processing techniques. This AI-based network sensing enhances cell search and supports handovers in multi-numerology cell-free environments, increasing the throughput and spectral efficiency of the 6G networks.

Together, these models form an important part of the backbone of an integrated AI-driven radio spectrum sensing chain for 6G. Combined with other modulation detection enhancements, they enable end-to-end inference of modulation type, waveform type, and numerology directly from raw IQ samples, a step toward the vision of an AI orchestrator, a unified framework capable of sensing, reasoning, and acting across all layers of the radio interface.

The main contributions of this paper are summarized as follows:

- AI-based waveform type detection: We design, implement, and evaluate a DL model that directly detects OFDM and OTFS waveforms from raw IQ samples. This approach removes the need for extra radio signal processing techniques.
- AI-based numerology scheme detection: We propose a new multitask AI model that identifies OFDM numerology parameters such as subcarrier spacing, FFT size, slot duration, and CP type, directly from IQ samples, changing numerology into an observable spectrum feature.

The rest of this article is organized as follows. Section 2 reviews the challenges of AI orchestration in 5G and future 6G networks. Section 3 presents the AI-based waveform type detector, describing the dataset generation process, the AI model design, and the results from training, validation, and evaluation. Section 4 presents the AI-based OFDM numerology scheme detector, detailing the dataset generation, the design of a multitask AI model, and the performance achieved in various parameter estimation tasks. Lastly, Section 5 discusses the wider implications of these results for AI-native orchestration in 6G and beyond.

## 2. Challenges of AI-Based Orchestration in 6G Networks

In the fifth generation of mobile communication several improvements have been implemented compared to LTE. The main improvements have addressed bandwidth efficiency, spectrum utilization, and RAN flexibility that can adapt to multiple communication scenarios.

One of the most significant advances of 5G is the increase in available bandwidth. In LTE networks, the maximum channel bandwidth is 20 MHz, while 5G can use bandwidths of up to 100 MHz in sub-6 GHz frequencies and up to 400 MHz in mmWave bands. The increase in communication channel bandwidth increased the data rates, bringing support for low-latency applications. In addition, the supported carrier frequencies have been extended from the LTE range, which was below 3.5 GHz, to include higher mid-band (3.5–7 GHz) and mmWave spectrum (24–100 GHz) [15]. This allowed 5G networks to deliver data rates exceeding 10 Gbps under controlled scenarios, compared to LTE’s typical maximum of around 1 Gbps [16]. 5G also introduced greater network resource flexibility through scalable numerology, with SCS ranging from 15 kHz (as in LTE) to 240 kHz, allowing operators to adapt deployments for different communication scenarios, which brought support for new use cases such as mMTC [17] URLLC and Enhanced Mobile Broadband (eMBB) [18]. Together, these improvements increased the 5G network capacity 100 times compared to LTE, allowed for latencies as low as 1 ms under favorable conditions, and support for up to one million devices per square kilometer.

In addition, the advances in UE positioning capabilities introduced through successive 3GPP releases have transformed 5G into a precise location ecosystem [19]. Beginning with Rel-16, which established sub-meter to meter-level accuracy targets for indoor and outdoor environments, subsequent enhancements in Rel-17 [20] and Rel-18 [21] expanded both the accuracy and flexibility of UE positioning. These include better Time Difference of Arrival (TDoA) and Angle of Arrival (AoA) methods, carrier-phase positioning (CPP) reaching sub-meter accuracy, and the inclusion of Sidelink [22] positioning to enhance localization in areas with limited coverage. In addition, the use of mmWave frequencies has enabled the development of Joint Communication and Sensing (JCS) and Integrated Sensing and Communication (ISAC) systems [23]. In these novel concepts, 5G waveforms are used not only to determine the position of active UEs, but also to detect the velocity and position of passive objects through radar-like sensing techniques based on the Doppler shift of OFDM subcarriers [24].

In parallel with improvements to the 3GPP standard in successive releases, the literature has shown interest in AI enhancement of the capabilities that can be achieved with 5G waveforms. For example, extensive research has been dedicated to improving user positioning by employing machine learning (ML) and DL models capable of learning complex nonlinearities in radio propagation. Fingerprinting-based approaches using CSI, Channel Impulse Response (CIR), or Reference Signal Received Power (RSRP) features combined with AI models such as Convolutional Neural Networks (CNNs), Long Short Term Memory (LSTMs), and autoencoders have achieved sub-meter accuracies indoors and meter-level accuracies outdoors, even under Non Line of Sight (NLOS) and multipath conditions [25,26,27,28,29,30,31,32,33]. Beyond user positioning, AI has also been used in spectrum sensing. Traditional likelihood-based detection is replaced by feature-driven deep learning algorithms that work directly on IQ samples [34], spectrograms [13], or constellation diagrams [35]. These approaches enable modulation detection, radio device fingerprinting, and anomaly detection in congested spectrum environments, with reported accuracies exceeding 90% at moderate SNR values. Adversarial autoencoders [36], predictive networks like PredNet [37], and CNN-based semantic segmentation frameworks [13] have shown the ability to detect spectrum anomalies and differentiate LTE/5G transmissions.

Recent works have also evaluated AI-driven physical layer optimization. This includes Neural Network (NN) based channel estimation [38], CSI compression with autoencoders [39], and DL-based beam selection [40]. These techniques reduce the computation overhead, improve communication channel feedback, and speed up beam selection in massive MIMO systems. These developments show the feasibility of AI techniques in 5G networks, as they are not limited to user positioning enhancement and sensing capabilities, but also enable adaptive spectrum management, anomaly detection, and optimized PHY-layer operations.

Currently, 3GPP has conducted several dedicated studies on the application of AI/ML in 5G. One of these studies focuses on the air interface (TR 38.843, Rel-18) [41] and the other on mobility (TR 38.744, Rel-19) [42]. TR 38.843 evaluates the use of AI/ML techniques for PHY-layer enhancement, with representative use cases including CSI feedback enhancement, beam management, and user positioning accuracy improvements. The results show that AI and ML-based CSI compression and prediction methods can cut computation overhead by up to 70%. They achieve this while keeping or improving reconstruction accuracy compared to traditional quantization methods. Similarly, AI-based beam prediction reduces latency and improves robustness in dynamic environments, and positioning enhancements achieve sub-meter accuracy in challenging NLOS scenarios.

TR 38.744 examines the role of AI/ML in mobility management. The study considers use cases such as Radio Resource Management (RRM) measurement prediction, measurement event prediction, and Radio Link Failure (RLF) prediction. Results show that AI/ML-based predictors can reduce measurement reporting computation load by exploiting temporal and spatial correlations, while improving handover decisions and reducing handoff failure (HOF) and RLF rates. System-level simulations confirm that AI-based mobility prediction contributes to smoother handovers and reduced ping-pong events.

In future 6G networks, AI techniques are expected to shift from a supportive role to AI-native architectures. In contrast to their role in 5G, where AI/ML techniques are used to enhance existing network functions, 6G is expected to embed AI techniques as a core element directly into the RAN. Building on the small improvements made in 5G, such as AI-assisted positioning, spectrum sensing, beam management, and mobility prediction, 6G aims to bring together communication, sensing, and computation under a single management layer. Operating at sub-THz and THz frequencies, 6G will deliver extremely high data rates. It will also support ISAC for environmentally aware networking, autonomous mobility, and digital twin applications. Key enablers include massive MIMO [43], Advanced Beamforming [44,45], Reconfigurable Intelligent Surfaces (RIS) [46], and cell-free architectures [47], where dense deployments of distributed APs jointly serve UEs without fixed cell boundaries, ensuring consistent quality of service (QoS) even in highly dynamic scenarios.

**Figure 1:**
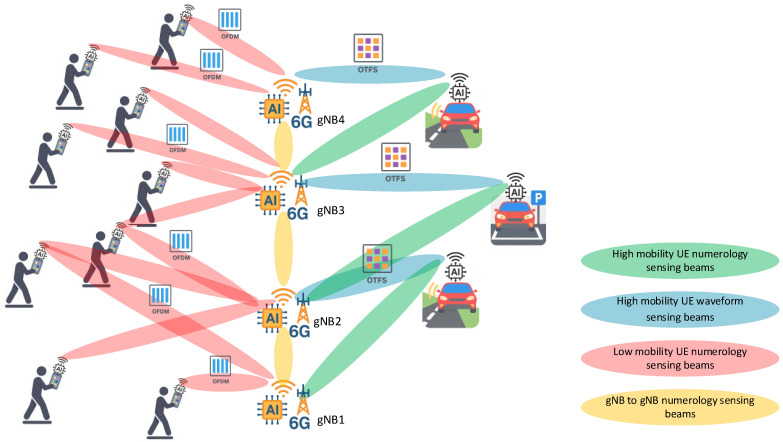
AI-orchestrated 6G gNBs coordinating sensing beams for heterogeneous UEs: low-mobility pedestrian UEs using OFDM, high-mobility vehicular UEs using OTFS, and inter-gNB numerology sensing.

In the context of 6G and beyond, spectrum sensing evolves from an unoccupied channel detection task into a comprehensive situational awareness capability. As shown in Figure 1, unlike older network topologies that depended on fixed, centrally managed waveforms, 6G imagines a mixed use of OFDM, OTFS, and other new multicarrier designs aimed at high mobility, very low latency, or fragmented spectrum.

Among the candidate waveform types for 6G, OFDM remains an effective choice for low-mobility and high-throughput situations because it makes efficient use of spectral resources and has a reliable receiver design. On the other hand, OTFS waveforms will be used for high-mobility scenarios. Its delay-Doppler domain representation offers strong resistance to time–frequency dispersion and quickly varying channel conditions. Additionally, new multicarrier formats and adaptive numerology schemes can take waveform flexibility even further. They support fragmented spectrum access and ultra-low-latency communication, which are needed for critical and machine-type applications.

In this ecosystem, different waveform types and numerology setups can exist in nearby cells or even within the same coverage area. AI-based spectrum sensing at the gNBs and UEs allows for identification of the active waveform and numerology from raw IQ samples. This supports waveform flexibility and quick changes in the receiver processing chains.

Waveform type sensing allows for waveform agility. This means radios can change their demodulation chain without requiring specific signaling. An AI-based waveform type detector allows this by enabling both gNBs and UEs to independently identify the received waveform type at the IQ level. In large-scale cell-free networks, waveform agility maintains high link reliability and maximizes spectral efficiency in crowded frequency bands. It also lays the groundwork for spectrum analysis. By monitoring the detected waveform and its parameters, the AI orchestrator can spot unusual patterns that may indicate interference, misconfigurations, or possible jamming attempts. This allows the development of mitigation strategies through dynamic guard band insertion, adaptive beam steering, or numerology adjustments. Besides the waveform type, extracting OFDM parameters like SCS, FFT size, slot duration, and CP type gives an overview of the numerologies active in the network. This supports AI-driven orchestration by allowing flexible scheduling and interference management in deployments with coexisting numerologies. An AI-based OFDM numerology detector that works directly with IQ samples makes this possible without higher-layer signaling. Integrated into a larger AI/RAN Intelligent Controller (RIC) orchestration framework, parameter awareness improves network reliability. It detects misconfigured UEs or gNB nodes and helps make spectrum allocation decisions.

This article introduces and evaluates two AI-based methodologies for 6G network orchestration. The first one aims to differentiate OFDM and OTFS waveforms, while the second one estimates OFDM numerology parameters directly from IQ data. Taken together with prior results on AI-driven modulation detection [12,48,49,50,51,52], these AI models can support the development of an integrated spectrum sensing chain, where modulation type, waveform type, and numerology are inferred. The following sections present the design, dataset generation, training, and evaluation of both AI models, highlighting their role as enablers of intelligent, cell-free, and adaptive 6G networks.

## 3. AI-Based Waveform Type Detector for 6G Networks

This section focuses on the detection of OFDM and OTFS waveforms as a spectrum sensing use case for AI-Orchestrated 6G networks. As presented in previous sections, waveform type detection represents a big part of the 6G network awareness ability. UEs and gNBs may need to switch between OFDM and OTFS depending on mobility, latency, or interference conditions. By detecting the waveform type directly from the received IQ samples, the receiver can select the required demodulation chain without depending on higher-level signaling from the network.

The first part of this work was the design of a dataset containing 10,080 OFDM and OTFS waveforms generated in the MATLAB environment using dedicated toolboxes [53,54]. Waveform numerologies were chosen based on HEXA documentation [55]. The resource block numbers were varied from 50 to 275. The SNR levels ranged from −4 dB to 30 dB. Each radio signal frame used 64-QAM, 256-QAM, 1024-QAM, or 4096-QAM constellations for modulation. After that, the power of each generated signal was normalized before adding channel impairments such as fading, frequency offsets, and Gaussian noise. Since the objective is waveform-type classification, pathloss attenuation was not directly modeled through the physical model, but by increasing the noise power relative to the normalized power of the signal. This ensures that classification performance depends on waveform-specific features rather than on received power variations. For OTFS waveform generation, the delay–Doppler grid was defined by $M=12N_{RB}$ subcarriers and N=14 OFDM symbols, resulting in grid dimensions ranging from 600×14 to 3300×14 depending on the selected resource block configuration. A rectangular transmit window with zero-padding equal to 25% of the symbol duration was used, matching Zero Padding (ZP)-type pulse shaping. This setup keeps inter-symbol interference low while maintaining the basic OTFS structure for waveform classification.

**Figure 2:**
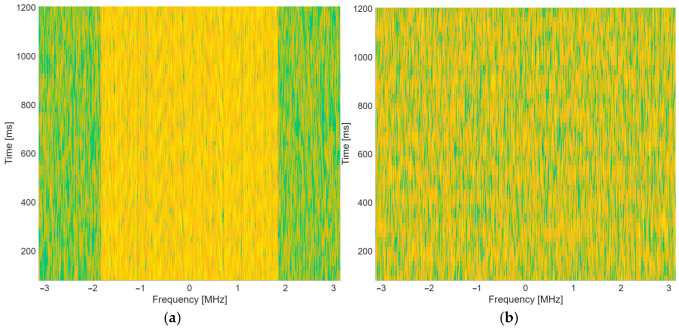
Dataset samples: (**a**) OFDM waveform; (**b**) OTFS waveform.

As presented in [48,56], emulated propagation effects and synthetic datasets can obtain good results in real-life inference scenarios. Figure 2 presents two spectrogram examples of dataset samples from both OFDM and OTFS waveforms.

Each waveform was cut or padded to 8192 IQ samples. This radio signal frame length provides sufficient temporal resolution for multiple symbols to be represented in the signal frame, while remaining computationally efficient from the perspective of the AI processing.

**Figure 3:**
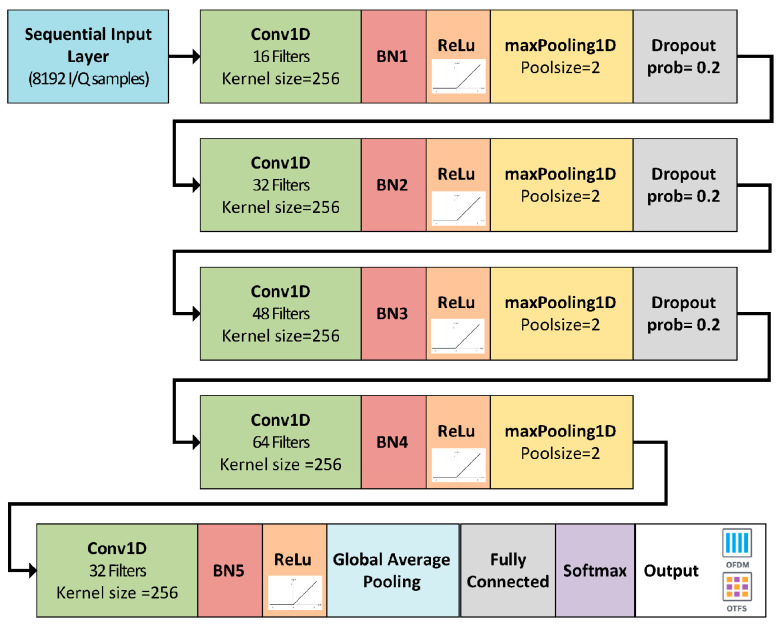
Waveform type detection AI model structure.

The AI model is a CNN structure that was adapted from our prior works on modulation recognition [48]. As presented in Figure 3, the network uses 8192 IQ samples as input and consists of a stack of five 1D convolutional blocks. In the first four blocks, the number of filters in the convolutional layers is increased, starting from 16 to 64 while maintaining a kernel size of 256. Each convolutional layer is followed by batch normalization, ReLU activation, max-pooling, and dropout with 0.2 probability. A fifth convolutional layer with 32 filters and a 256 kernel size is placed before a global average pooling layer, which compresses the temporal dimension into a compact representation. This is further passed through a fully connected layer, softmax activation, and finally mapped to the binary output classes (OFDM, OTFS). The relatively large convolutional kernels were chosen to better capture the dependencies and discriminative patterns of multicarrier waveforms. The network comprises approximately 1.85 million trainable parameters and requires about 1.4 GFLOPs per inference for an 8192 IQ sample input. The model was trained using stochastic gradient descent with momentum (SGDM), starting with a learning rate of 0.1 that decayed by a factor of 0.9 after each epoch, over a total of 100 epochs. The mini-batch size was set to 64, and after each training epoch, the dataset was shuffled and validation was performed in order to improve generalization. Hyperparameters were chosen based on extended empirical testing of the AI model. The model with the lowest validation loss was selected for final evaluation. A summary of the system setup and training parameters is provided in Table 1.

**Figure 4:**
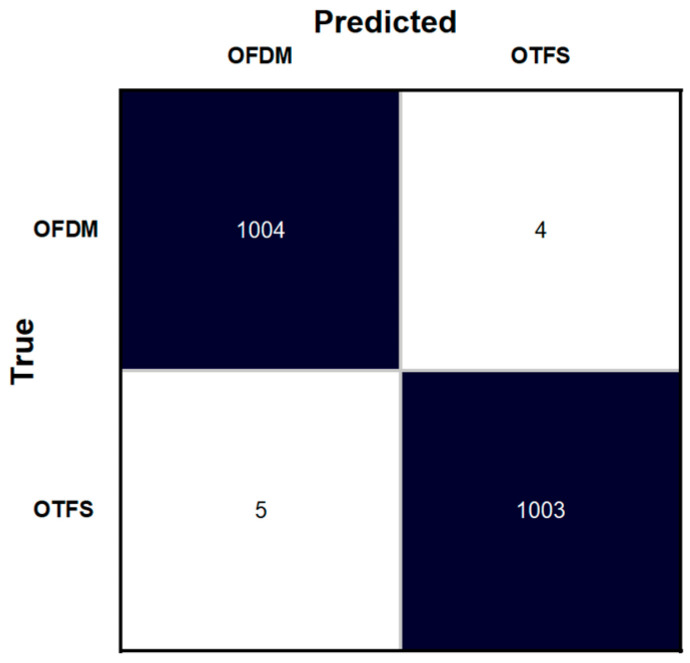
Confusion matrix for OFDM-OTFS waveform detector.

The trained AI model for waveform type detection achieved 98.4% accuracy on the validation dataset and 99.5% on the test set. Also, as seen in the confusion matrix presented in Figure 4, only 9 classification errors out of 2016. As shown in Table 2, the model achieved high performance metrics on the test dataset. It had a precision of 0.995, a recall of 0.996, a specificity of 0.995, and an F1 score of 0.9955. The advantage of this approach is that the waveform type detection is performed directly on IQ samples, without the need for pre-processing or explicit feature extraction. This enables fast adaptation of the demodulator chain to the detected waveform, supporting waveform agility. Additionally, waveform detection improves spectrum efficiency by reducing the resource blocks used for network signaling, which can be used for data exchange between UEs and gNBs.

## 4. AI-Based OFDM Numerology Detector

In addition to waveform type classification, future 6G UEs and gNBs will require the ability to detect key parameters of the used waveforms—in the case of cellular communication, the OFDM waveform—without relying only on high-layer signaling. In current 5G systems, the establishment of a connection between a UE and a gNB involves several well-defined steps, beginning with synchronization to the Synchronization Signal Blocks (SSBs), followed by the random access procedure, Radio Resource Control (RRC) configuration, authentication, and ultimately the setup of PDU sessions. The gNB configures subcarrier spacing, slot duration, and cyclic prefix type to meet the communication scenario demands. Meanwhile, the UE receives this information from higher-layer signaling. However, an AI-based OFDM numerology detector can extract these parameters directly from IQ samples. This can speed up access, lower signaling overhead, and allow changes in dynamic multi-numerology environments.

Beyond the initial access procedure, parameter inference provides additional benefits for network optimization. The ability to detect numerology mismatches between neighboring gNBs or APs in cell-free networks lets schedulers insert guard bands, set slot boundaries, or adjust beam patterns in order to reduce inter-numerology interference. In mobility scenarios, a UE that can anticipate the target cell’s parameters prior to handover will experience reduced latency and improved service continuity. At the network level, feeding AI-inferred parameters into a RAN Intelligent Controller (RIC) [57] enables real-time analytics and supports troubleshooting of misconfigured nodes. This helps create a more secure and efficient 6G network.

To validate this concept, a large dataset composed of 36,000 OFDM waveforms was generated using the MATLAB dedicated LTE and 5G toolboxes [54,58], under a wide range of numerologies and channel conditions. Downlink signals were created for both LTE and 5G NR, with NR spanning subcarrier spacings of 15, 30, 60, and 120 kHz, and LTE fixed at 15 kHz. Both normal and extended cyclic prefixes were included. FFT sizes and slot durations were chosen based on the numerology. Each frame was modulated using modulation schemes from QPSK to 1024-QAM. As in the previous dataset, emulated propagation effects were added to the generated signals. The dataset was randomly split into training (60%), validation (20%), and testing (20%) subsets. The class imbalance in the training process was accounted for using inverse frequency weights for each class and classification task. To improve robustness, light augmentation was applied during training in the form of time shifts and small IQ rotations, helping the model generalize to timing offsets and carrier-phase variability.

**Figure 5:**
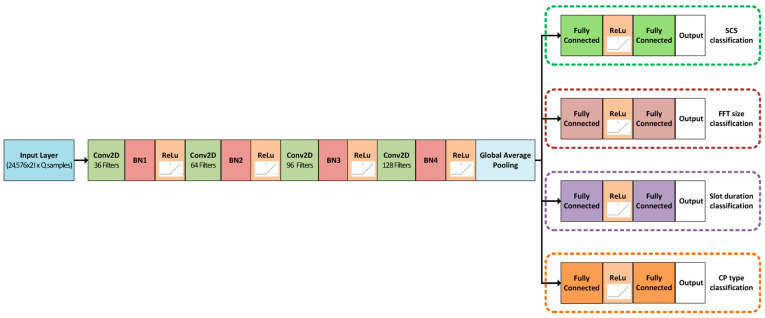
Multitask OFDM waveform parameter detection AI model structure.

As presented in Figure 5 for OFDM numerology detection, a compact CNN was designed to process the IQ sequences as two-channel time series. The backbone consisted of a sequence of temporal convolution layers with progressively increasing depth and downsampling, each followed by batch normalization and nonlinear activation. A global average pooling layer then compresses the temporal dimension, producing a compact representation of the received signal. As shown in Figure 5, the OFDM numerology detector employs four convolutional blocks, followed by global average pooling, and four task-specific classification heads for predicting the SCS, FFT size, slot duration, and CP type. The network contains approximately 0.18 million trainable parameters and requires about 0.14 GFLOPs per inference for a 24,576-sample IQ input. A summarization of the hardware setup, dataset size and training parameters is presented in Table 3.

The training process used a weighted combination of cross-entropy losses across the four tasks. Each classification head is optimized using the loss function as shown in (1), where h∈{SCS,FFT,Slot,CP} indexes the task head, B denotes the mini-batch size, $C_{h}$ is the number of classes for the task h, $Z_{c,i}^{\left(h\right)}$ is the logit output corresponding to class c of sample i for head h, and $y_{i}^{\left(h\right)}$ is the ground-truth class index of sample i in task h. The term $w_{y_{i}^{\left(h\right)}}^{\left(h\right)}$ is used to mitigate class imbalance, representing the normalized inverse frequency weight associated with the true class label, and as presented in (2), is computed using $p_{c}^{\left(h\right)}$ representing the occurrence of class c for task head h in the training dataset. The ε term is a small constant equal to $10^{-7}$ added for numerical stability inside the logarithm. Finally, the overall loss L for the training process is computed according to (3) as the sum of each task head loss $L_{h}$ multiplied by $\lambda_{h}$, which represents the task weight, equal to 0.25, as all parameters were considered equally important for OFDM parameter identification. During training, the Adam optimizer was used, with a learning rate of 0.001, mini-batch size of 64, and 100 training epochs, with gradient clipping to stabilize network updates.

(1) $$L_{h}=\frac{1}{B}\sum_{i=1}^{B}w_{y_{i}^{\left(h\right)}}^{\left(h\right)}(-\log\frac{\exp(Z_{y_{i}^{\left(h\right)},i}^{\left(h\right)})}{\sum_{c=1}^{C_{h}}\exp(Z_{c,i}^{\left(h\right)})+\varepsilon }).$$

(2) $$w_{c}^{\left(h\right)}=\frac{\frac{1}{p_{c}^{\left(h\right)}}}{\sum_{k=1}^{C_{h}}\left(\frac{1}{p_{c}^{\left(h\right)}}\right)}.$$

(3) $$L=\sum_{h\in \{SCS,FFT,Slot,CP\}}\lambda_{h}L_{h},\;\;\lambda_{h}=0.25.$$

From the performance metrics presented in Table 4 we can see that the model achieved a high level of performance across all four estimation tasks. On the test set, accuracies reached 99.75% for subcarrier spacing, 99.9% for FFT size, 96.7% for slot duration, and 83.3% for cyclic prefix (CP) type, with corresponding F1 scores of 0.999, 0.999, 0.964, and 0.866, respectively. The results show a trade-off between recall and precision across classification tasks. For example, CP type estimation achieved high precision (0.9982) and specificity (0.9968), despite lower recall (0.7639). The joint accuracy, defined as all four parameters being correctly identified simultaneously, reached 80% indicating a high level of performance of the proposed method in multi-parameter estimation scenarios.

**Figure 6:**
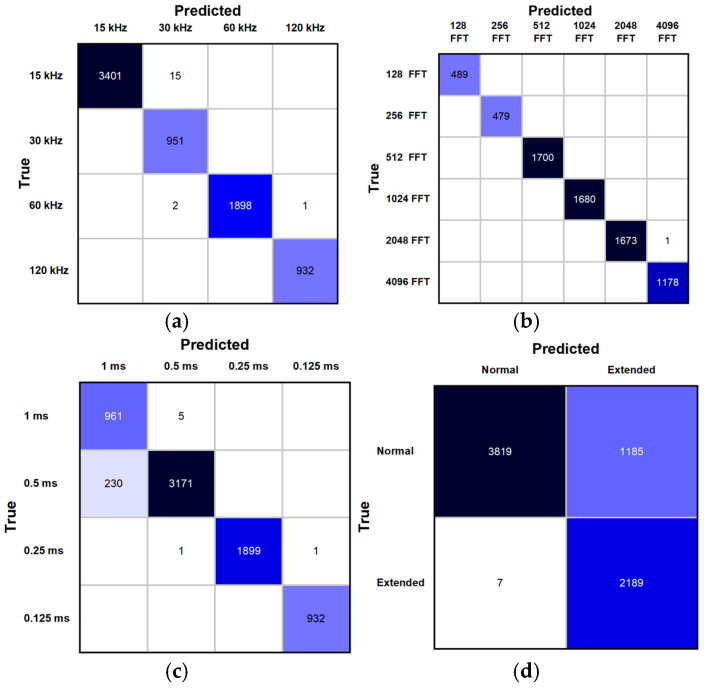
Confusion matrices for OFDM numerology detector: (**a**) Sub-carrier Spacing; (**b**) FFT Size; (**c**) Slot Duration; (**d**) Cyclic Prefix type.

Confusion matrices presented in Figure 6 show that subcarrier spacing and FFT size classification have a small amount of errors, while slot duration classification errors were mostly limited to confusion between adjacent values. In contrast, CP type misclassifications primarily showed the similarity between extended and normal CP under noisy conditions. These results confirm that numerology parameters can be inferred directly from raw IQ samples with high accuracy, bypassing the need for higher-layer signaling or additional signal processing.

Once OFDM parameters are recovered, the symbols can be reconstructed and subsequently passed to AI-based modulation classifiers, as demonstrated in prior works [12,48,49,50,51,52]. The addition of modulation recognition creates a more complete sensing pipeline. In this setup, we can infer waveform type, OFDM numerology parameters, and modulation order from the same IQ stream. This integrated framework simplifies receiver design. It also gives an AI-driven orchestrator a clearer view of the radio environment. The achieved PHY-layer awareness allows for smart scheduling decisions, better resource allocation in cell-free deployments, and increased resilience against interference, challenging conditions, or network misconfigurations. Additionally, inferring numerology parameters allows for numerology flexibility in 6G networks. In this setup, user equipment and gNBs can change subcarrier spacing, slot duration, or cyclic prefix based on the communication situation.

## 5. Discussion

The transition from 5G to 6G marks a change from AI-assisted optimization to AI-native orchestration. In this new phase, intelligence is a core feature of the RAN instead of just a network enhancement technique. Unlike earlier generations that depended on preset signaling and fixed setups, 6G networks need to dynamically adjust across different deployment scenarios, varied sub-THz frequency ranges, and high-mobility scenarios like UAVs, V2X networks, and satellite connections.

This vision of future mobile networks requires radio units that can sense the radio spectrum, reason about contextual information, and adapt. These capabilities are enabled through an AI orchestration layer that unifies sensing, learning, and decision-making across all network domains. Within this context, the present work introduces two AI-based detectors that support spectrum awareness and waveform agility.

The first detector addresses the waveform-type identification. While OFDM is still the standard waveform for cellular systems, OTFS is becoming popular as a candidate for 6G, especially in high-mobility communication scenarios. In this way, both UEs and gNBs can detect if the received waveform is OFDM or OTFS type. Differentiating between the waveform types gives receivers the ability to choose the right demodulation chain without depending on higher-layer signaling or additional signal processing.

Using a CNN trained on over 10,000 signals, the waveform-type detector achieved a classification accuracy of 99.5%. These results show that deep learning models can effectively capture spectral-temporal relationships of multicarrier waveforms directly from IQ data. The second contribution is an AI-based OFDM numerology detector that enhances spectrum awareness by determining physical-layer parameters that define the OFDM waveform structure. Unlike current methods that require higher-layer signaling or signal processing to determine subcarrier spacing, FFT size, slot duration, and cyclic prefix, this detector estimates these parameters directly from received IQ samples.

Trained on a dataset of 36,000 LTE and NR signals, the model achieved accuracies of 99.9% for FFT size and subcarrier spacing, 96.7% for slot duration, and 83.3% for cyclic prefix type, with an overall multi-parameter accuracy of 80%. Accurate cyclic prefix detection was more difficult to achieve due to temporal similarities in noisy conditions.

By converting waveform types and numerology schemes from fixed setups into observable features, the proposed AI-based detectors lay the groundwork for AI orchestration in future 6G networks. These capabilities lower synchronization failures during initial access, speed up UE network attachment, and improve handover reliability in multi-numerology, cell-free deployments.

From a methodological viewpoint, the main advantage of the proposed approach is its ability to process raw IQ samples directly, eliminating the need for further signal processing prior to detection. Additionally, using multitask architectures, like the numerology detector, shows that deep learning can manage multi-dimensional PHY-layer inference effectively.

Nonetheless, several limitations should be addressed in future work. Firstly, since this work is presented as a proof of concept, the models were trained and validated on synthetic datasets under controlled channel conditions. The generated datasets contain a wide range of SNR values, signal parameters, and channel impairments. However, they cannot fully capture real-world propagation effects such as hardware imperfections, nonlinearities, and communication scenario variations. Therefore, future use of over-the-air capture datasets should be considered. Second, while the proposed CNNs can be handled easily in offline settings, they may create problems for real-time edge processing. These situations can occur in crowded deployments or for devices with limited power. Considering this, lightweight designs, pruning, or quantization will be considered in the future. Third, while the AI models achieved high accuracy for most tasks, cyclic prefix detection remained relatively low (~83%), suggesting that subtle temporal structures are harder to detect under noisy conditions. Hybrid approaches that combine AI-based inference with lightweight signal processing can be considered for future implementations. Finally, this work is focused on PHY-layer awareness. The cooperation between PHY-layer AI detectors and upper-layer orchestration mechanisms, such as RRM, beam management, or mobility prediction, remains an open area for cross-layer optimization.

In summary, this work demonstrates that AI-based spectrum awareness for future 6G networks is feasible, obtaining accuracies above 90% for most of the considered tasks. The results validate the use of AI techniques for PHY-layer spectrum sensing and AI-native orchestration in 6G networks.

## Figures and Tables

**Table 1:** System setup and AI model training parameters for the waveform type detector.

Parameter	Specification
Processor	AMD Ryzen 9 3950X, 16 cores
System Memory	64 GB RAM
GPUs	2 × NVIDIA GeForce RTX 3060
Software Environment	MATLAB
Dataset Size	10,080 waveforms (OFDM and OTFS)
Input Length	8192 IQ samples
Optimizer	SGDM
Initial Learning Rate	0.1 (decayed by 0.9 per epoch)
Batch Size	64
Epochs	100
Trainable Parameters	≈1.85 million
Computational Cost	≈1.4 GFLOPs per inference

**Table 2:** Performance metrics of the Waveform-type detector AI model.

Precision	Recall	Specificity	F1 Score
0.995	0.996	0.995	0.9955

**Table 3:** System setup and AI model training parameters for OFDM numerology detector.

Parameter	Specification
Processor	AMD Ryzen 9 3950X, 16 cores
System Memory	64 GB RAM
GPUs	2 × NVIDIA GeForce RTX 3060
Software Environment	MATLAB
Dataset Size	36,000 OFDM waveforms (LTE and NR)
Input Length	24,576 IQ samples
Optimizer	Adam
Initial Learning Rate	0.001 (fixed)
Batch Size	64
Epochs	100
Trainable Parameters	≈0.18 million
Computational Cost	≈0.14 GFLOPs per inference

**Table 4:** Performance metrics of the OFDM numerology detector AI model.

Classification Task	Accuracy	Precision	Recall	Specificity	F1 Score
Subcarrier Spacing	0.9975	0.999	0.999	0.999	0.999
FFT Size	0.999	0.999	0.999	0.999	0.999
Slot duration	0.967	0.951	0.982	0.99	0.964
CP Type	0.833	0.9982	0.7639	0.9968	0.8656

## Data Availability

The datasets presented in this article are available on request from the authors.

## Abbreviations

4G	Fourth Generation (mobile communication system)
5G	Fifth Generation (mobile communication system)
6G	Sixth Generation (future mobile communication system)
AI	Artificial Intelligence
CIR	Channel Impulse Response
CNN	Convolutional Neural Network
CP	Cyclic Prefix
CPP	Carrier-Phase Positioning
CSI	Channel State Information
DL	Deep Learning
eMBB	Enhanced Mobile Broadband
FFT	Fast Fourier Transform
FR	Frequency Range
gNB	gNodeB (Next Generation Base Station in 5G/6G)
HOF	Handoff Failure
IQ	In-phase and Quadrature (signal components)
ISAC	Integrated Sensing and Communication
JCS	Joint Communication and Sensing
LEO	Low Earth Orbit
LTE	Long-Term Evolution (4G)
MIMO	Multiple Input Multiple Output
ML	Machine Learning
mMTC	Massive Machine Type Communications
NLOS	Non-Line of Sight
NN	Neural Network
NR	New Radio (5G standard)
OFDM	Orthogonal Frequency Division Multiplexing
OTFS	Orthogonal Time Frequency Space
PDU	Protocol Data Unit
PHY	Physical Layer
QAM	Quadrature Amplitude Modulation
QoS	Quality of Service
QPSK	Quadrature Phase Shift Keying
RAN	Radio Access Network
RB	Resource Block
RIC	RAN Intelligent Controller
RIS	Reconfigurable Intelligent Surface
RLF	Radio Link Failure
RRC	Radio Resource Control
RRM	Radio Resource Management
RSRP	Reference Signal Received Power
SCS	Subcarrier Spacing
SGDM	Stochastic Gradient Descent with Momentum
SNR	Signal-to-Noise Ratio
UAV	Unmanned Aerial Vehicle
UE	User Equipment
URLLC	Ultra-Reliable Low-Latency Communication
ZP	Zero Padding
